# Analysis of Enzyme Activity and Cellular Function for the N80S and S480F Asparagine Synthetase Variants Expressed in a Child with Asparagine Synthetase Deficiency

**DOI:** 10.3390/ijms24010559

**Published:** 2022-12-29

**Authors:** Stephen J. Staklinski, Sarah Snanoudj, Anne-Marie Guerrot, Catherine Vanhulle, François Lecoquierre, Soumeya Bekri, Michael S. Kilberg

**Affiliations:** 1 Department of Biochemistry and Molecular Biology, University of Florida College of Medicine, Gainesville, FL 32610, USA; 2 School of Biological Sciences, Cold Spring Harbor Laboratory, Cold Spring Harbor, NY 11724, USA; 3 Department of Metabolic Biochemistry, Rouen University Hospital, F-76000 Rouen, France; 4 University Normandie UNIROUEN, CHU Rouen, INSERM U1245, F-76000 Rouen, France; 5 Department of Genetics and Reference Center for Developmental Disorders, Rouen University Hospital, F-76000 Rouen, France; 6 Department of Neonatalogy, Pediatric Intensive Care and Neuropediatrics, Rouen University Hospital, F-76000 Rouen, France

**Keywords:** amino acid, metabolism, cell proliferation, brain metabolism, inborn error of metabolism, epilepsy

## Abstract

Asparagine Synthetase Deficiency (ASNSD) is a disease caused by mutations in asparagine synthetase (*ASNS*). Newborns exhibit microcephaly, intractable epileptic-like seizures, progressive brain atrophy, and axial hypotonia. ASNSD results in global developmental delays and premature death. The present report describes a 9-year-old child who is a compound heterozygote with *ASNS* mutations c.1439C > T and c.239A > G leading to variants p.S480F and p.N80S, respectively. When grown in a complete culture medium, primary fibroblasts from the child contained ASNS mRNA and protein levels similar to an unrelated wild-type fibroblast cell line. When the child’s fibroblasts were cultured for up to 72 h in a medium lacking asparagine, proliferation was reduced by about 50%. Purification of ASNS proteins harboring either the S480F or the N80S substitution had reduced enzymatic activity by 80% and 50%, respectively. Ectopic expression of either variant in ASNS-null Jensen rat sarcoma (JRS) cells did not support proliferation in the absence of medium-supplied asparagine, whereas expression of wild-type enzyme completely restored growth. These studies add to the list of pathogenic ASNS variants and use enzyme activity and protein expression in ASNS-null cells to expand our knowledge of the biological impact of mutations in the *ASNS* gene.

## 1. Introduction

Asparagine synthetase (ASNS) catalyzes the de novo synthesis of asparagine (Asn) from substrates glutamine (Gln) and aspartate (Asp) in an ATP-dependent amidotransferase reaction [1]. The human *ASNS* gene is comprised of 13 exons within 35 kb at chromosome 7 region 7q21.3 [2,3,4]. The *ASNS* gene is regulated by a variety of cell stresses, such as amino acid deprivation and ER stress [5,6]. The protein contains 561 amino acids and has a mass of 64 kDa. The human ASNS crystal structure has been reported (PDB: 6GQ3) [7] and shown to be homologous with *E. coli* asparagine synthetase B (PDB: 1CT9) [1,8]. Residues 209–561 within the C-terminal domain contain the ATP and Asp binding sites, and this domain catalyzes the activation of the Asp carboxyl group by an ATP-dependent process to generate an enzyme-bound β-aspartyl-AMP intermediate. Residues 1–208, which make up an N-terminal domain, bind Gln such that the carboxamide group is directed toward the C-terminal domain, and the glutaminase activity generates an ammonia group. This ammonia is thought to diffuse through an intramolecular channel to react with the β-aspartyl-AMP intermediate, producing Asn [1,7,8,9].

Asparagine synthetase deficiency (ASNSD) was initially reported in 2013 [10], and since then, about 75 patients within more than 50 families have been described following whole exome sequencing to identify biallelic homozygous or compound heterozygous mutations in the *ASNS* gene [11]. Children with ASNSD typically display microcephaly, intractable seizures, progressive brain atrophy, encephalopathy, appendicular spasticity, and axial hypotonia [10,12,13]. The mechanistic basis for these symptoms is not fully understood, but the observations that cells from ASNSD children rely on extracellular Asn for proliferation [14,15], variant ASNS proteins have reduced Asn synthesis activity [7,16,17] or cannot be expressed due to instability [10,17], and that variant ASNS proteins cannot rescue growth of an ASNS null cell line [16,17] all indicate a direct link between the variants and ASNS enzymatic deficiency. It is hypothesized that the neurological phenotype results from the known function of the blood–brain barrier to utilize transporters on the two surfaces of polarized endothelial cells to mediate the net efflux of Asn [18] and, thus, rely on intracellular Asn synthesis by ASNS [6].

The current report describes analysis of fibroblasts of an ASNSD child who expresses p.Asn80Ser (N80S) and p.Ser480Phe (S480F) variant ASNS proteins. The child presented in the clinic with progressive epileptic encephalopathy, severe developmental delay, microcephaly, and cortical blindness. Using child and maternal primary dermal fibroblasts as a model, proliferation in the absence of extracellular Asn and the endogenous ASNS mRNA and protein levels were determined. The N80S and S480F variants were expressed, purified, and assayed for activity and thermal stability. Both variants were also expressed in an ASNS-null genetic background cell line to evaluate the ability of each variant, independent of other alleles, to sustain growth in an environment necessitating Asn synthesis.

## 2. Results

### 2.1. Diagnosis and Clinical Assessment

**Figure 1:**
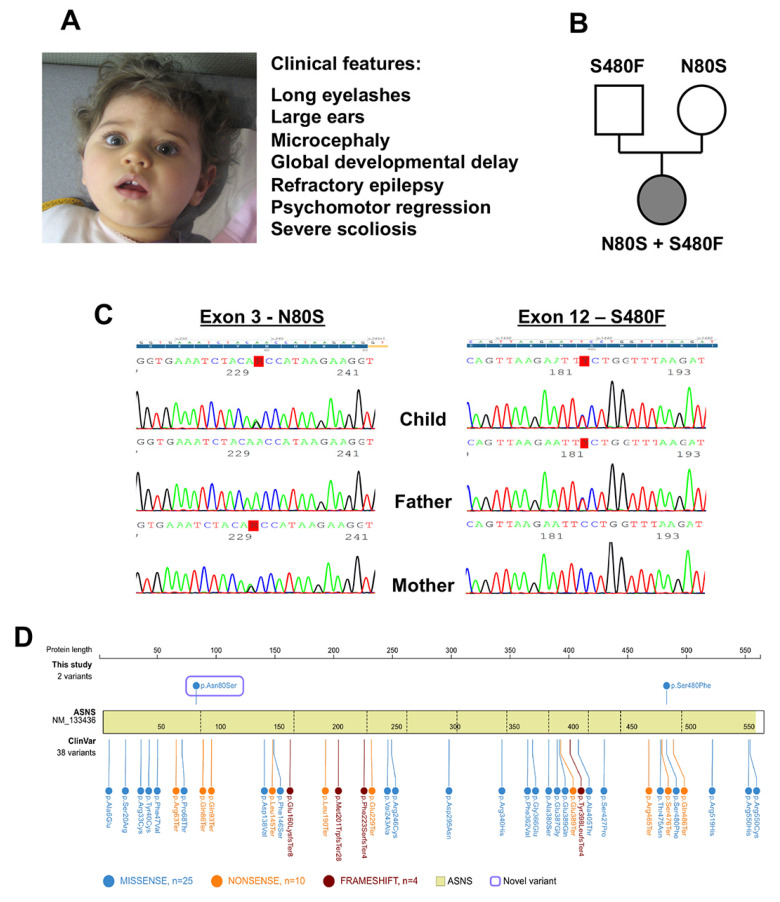
Clinical and genetic features of the affected child. (**A**) Clinical observations. (**B**) Family pedigree of the present family. (**C**) Sanger sequencing results. “Y” represents paternal c.1439C > T, and “R” represents maternal c.239A > G. (**D**) The identified *ASNS* variants in the present report, along with the 38 pathogenic or probably pathogenic exonic variants described in ClinVar.

The child was born at full term from unrelated and healthy Caucasian parents. The absence of ocular following was first noted by the mother at 3 mo of age. At 8 mo of age, the child presented with weight stagnation, absence of eye contact, axial hypotonia, and delayed neurodevelopmental milestones. Intractable epilepsy appeared at 14 mo. Her neurodevelopment continued to slowly progress with intensive physiotherapy until about 2 years of age, at which time it halted, and then she subsequently regressed. Currently, at age 9, she has an epileptic encephalopathy, severe microcephaly over -4 SD, and cortical blindness; she does not react to her name, has spontaneous flexion of all 4 limbs, moves to extension when touched, and is spastic. She has an abnormal sleep pattern and is exclusively fed by gastrostomy. She has severe scoliosis (Figure 1A).

The evaluation showed normal visual-evoked potential test and fundus, with slow activity compared to age-related standards on EEG. A thin corpus callosum associated with large peri-cerebral spaces was observed by cerebral MRI. The metabolic assessment showed repeated elevated blood lactate (up to 3.20 mmol/L, normal range 0.50–2.20) and lactate/pyruvate ratio (up to 34, N < 20), but normal lactate concentration in the CSF and normal blood amino acid concentrations. The rest of the child’s assessment was unremarkable.

The genetic analysis revealed a 68 kb duplication at 4q33 inherited from the healthy father (a variant of unknown significance) on the Comparative Genomic Hybridization (CGH)-array. The search for Angelman and Rett syndrome was negative. Multiple Next-Generation Sequencing studies for microcephaly, intellectual disability, and lysosomal diseases were performed without molecular diagnosis. Whole-exome sequencing showed two composite heterozygous mutations in *ASNS* (NM_133436.3): c.1439C > T (p.S480F) (in exon 12, genomic position Chr7:g.97482409G > A), inherited from the father, which has already been reported and was classified as probably pathogenic (class IV according to the ACMG guidelines) [19], and c.239A > G (p.N80S) (in exon 3, genomic position Chr7:g.97498230T > C), inherited from the mother, a variant of unknown significance (Figure 1B,C). Both variants were predicted to be disease causing by Mutation Taster, probably damaged by PolyPhen-2, and deleterious by PROVEAN. Given these genetic results, along with the child’s presentation, ASNSD was strongly suspected. Figure 1D shows the identified *ASNS* variants in the present report, along with 38 pathogenic or probably pathogenic exonic variants described in ClinVar.

### 2.2. Computer Modeling of N80S and S480F ASNS Variants

**Figure 2:**
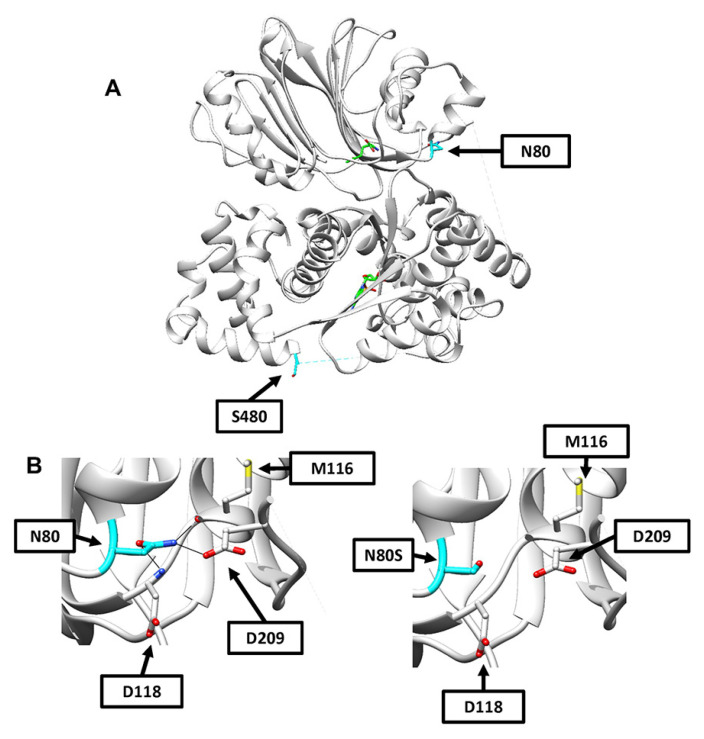
Computational models of N80S and S480F variants. (**A**) A model of the entire human ASNS crystal structure showing the locations of N80 and S480 in cyan. Glutamine is shown in the N-terminal binding site and AMP in the C-terminal binding site, with both backbones in green, nitrogen atoms in blue, oxygen atoms in red, and phosphorous in orange. (**B**) Shown in the (**left**) panel is a close-up of the region containing N80 and showing predicted hydrogen bonding interactions with M116 (2.9 angstroms), D209 (2.9 angstroms), and D118 (2.9 angstroms). Shown in the (**right**) panel is the same region now displayed with the most probable N80S rotamer in place. Increased distances lead to the absence of hydrogen bonding predictions between N80S and M116 (3.9 angstroms), D209 (3.9 angstroms), and D118 (3.6 angstroms).

Overlaying the human ASNS crystal structure (PDB 6GQ3) [7] with glutamine bound in the N-terminal domain onto the *E. Coli* AS-B crystal structure (PDB 1CT9) which had AMP bound in the C-terminal domain, computational models were developed to predict the functional consequence of the N80S and S480F variants. N80S is a novel variant not previously reported that is located within the N-terminal of the protein but not near the glutaminase active site (Figure 2A). N80 is predicted to hydrogen bond with residues M116 (2.9 angstroms), D118 (2.9 angstroms), and D209 (2.9 angstroms) (Figure 2B, left panel). When the most probable N80S rotamer is substituted, these bonds are likely lost due to their respective lengths increasing to 3.9, 3.6, and 3.9 angstroms (Figure 2B, right panel). No steric interference was predicted for the N80S residue, although the shorter side chain may impact folding within the nearby chain, which is located near the surface of the protein. The S480F variant has been previously reported in another patient [19], and we analyzed the possible structural implications based on the *E. coli* AS-B crystal, which suggested that the introduction of a nonpolar residue on the protein surface may impact protein solubility [6]. Current modeling using the human ASNS structure supports the earlier prediction, as the most probable S480F rotamer is not predicted to hydrogen bond with local residues or lead to steric interference (Figure 2A).

### 2.3. Endogenous ASNS mRNA and Protein Levels in Primary Fibroblasts

**Figure 3:**
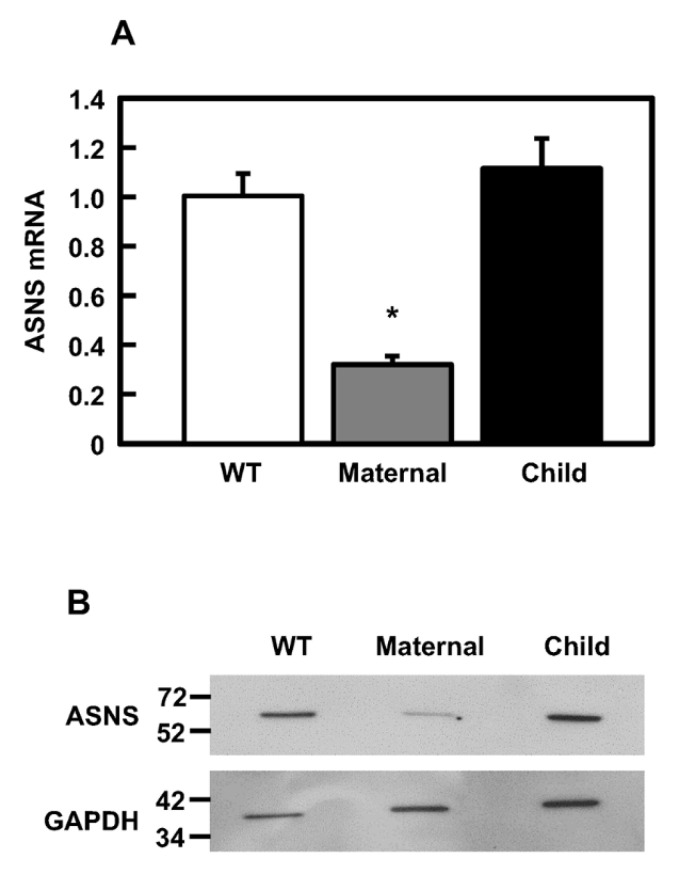
ASNS mRNA and protein evaluation in fibroblasts. (**A**) ASNS mRNA abundance, normalized to GAPDH mRNA and plotted relative to WT, as detected by qRT-PCR for WT, maternal, and child fibroblasts. An asterisk indicates that the value is statistically different from the WT, with a *p*-value of ≤0.05. (**B**) Immunoblot for ASNS protein in WT, maternal, and child fibroblasts.

The mRNA and protein levels for ASNS were determined in cultured fibroblasts from an unrelated WT, the affected child, and the mother to provide context for further patient cell characterization. A skin biopsy from the father could not be obtained. The *ASNS* mRNA abundance in the WT and child-derived cells was similar, whereas the maternal line exhibited about a 60% decrease in ASNS mRNA (Figure 3A). The mRNA results were reflected in the relative ASNS protein abundance as measured by immunoblotting (Figure 3B). The reason for the decreased ASNS mRNA and protein content in the cells from the heterozygotic mother is not known.

### 2.4. Enzyme Activity of Purified WT, N80S, and S480F ASNS Protein

**Figure 4:**
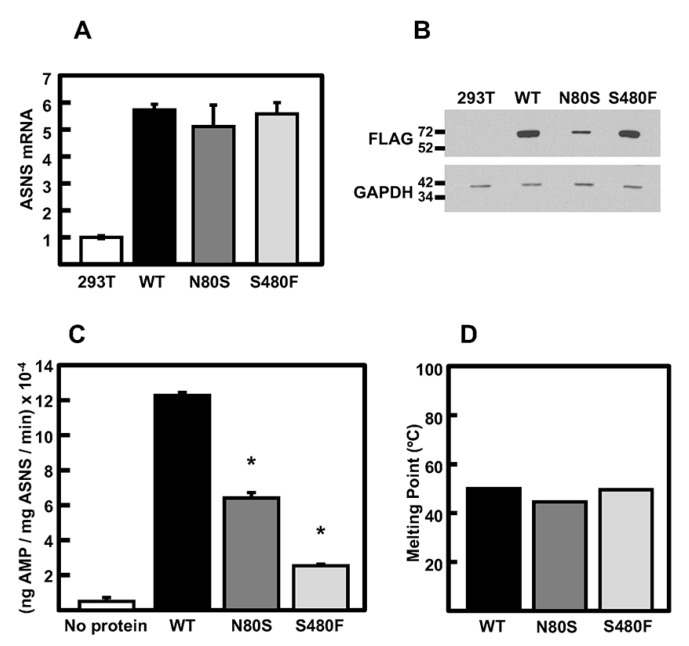
Enzyme activity and thermal stability of purified ASNS proteins. (**A**) ASNS mRNA levels in non-transfected HEK293T cells and in HEK293T-based cell lines stable-expressing WT, N80S, or S480F ASNS variants. The mRNA content was detected by qRT-PCR, normalized to GAPDH mRNA, and plotted relative to WT. (**B**) Immunoblot for each of the ASNS variant proteins. GAPDH protein served as the loading control. (**C**) Enzyme activity of purified WT, N80S, and S480F ASNS proteins. A reaction with no protein added serves as a negative control to show background detection. The data are the averages ± standard deviations of assays in triplicate, and an asterisk indicates that the value is statistically different from the WT with a *p*-value of ≤0.05. (**D**) The melting points of purified WT, N80S, and S480F proteins as detected by differential scanning fluorimetry.

Ectopic expression in HEK293T cells and subsequent purification of FLAG-tagged N80S and S480F variants together with WT ASNS were performed to analyze enzymatic activity. Asparagine production by purified ASNS protein variants has been reported using HPLC [16], but direct analysis of asparagine production has proven difficult for many laboratories. To assay ASNS enzymatic activity, we initially attempted analysis of pyrophosphate production as reported by Zhu et al. [7], but we were unsuccessful in obtaining reproducible results. However, using a commercially available kit, we were able to consistently analyze AMP production by purifying ASNS protein. Both variants exhibited mRNA abundance that was similar to the WT construct (Figure 4A). The protein abundance of the S480F variant was the same as that for the WT, but the steady-state abundance of the N80S protein was clearly reduced (Figure 4B), a result that may suggest some protein instability. Each protein was purified using a FLAG affinity column, and the enzymatic activity was assayed with saturating substrate conditions, which revealed a nearly 50% decreased activity for N80S and 80% decreased activity for S480F relative to the WT protein (Figure 4C). Analysis of the purified proteins by differential scanning fluorimetry demonstrated similar melting points for all three proteins (Figure 4D). Although the modeling did not predict major changes in protein structure for each of the variants, these enzymatic data indicate a clear deficiency in the function of both.

### 2.5. Proliferation of Fibroblasts in the Absence of Extracellular Asparagine

**Figure 5:**
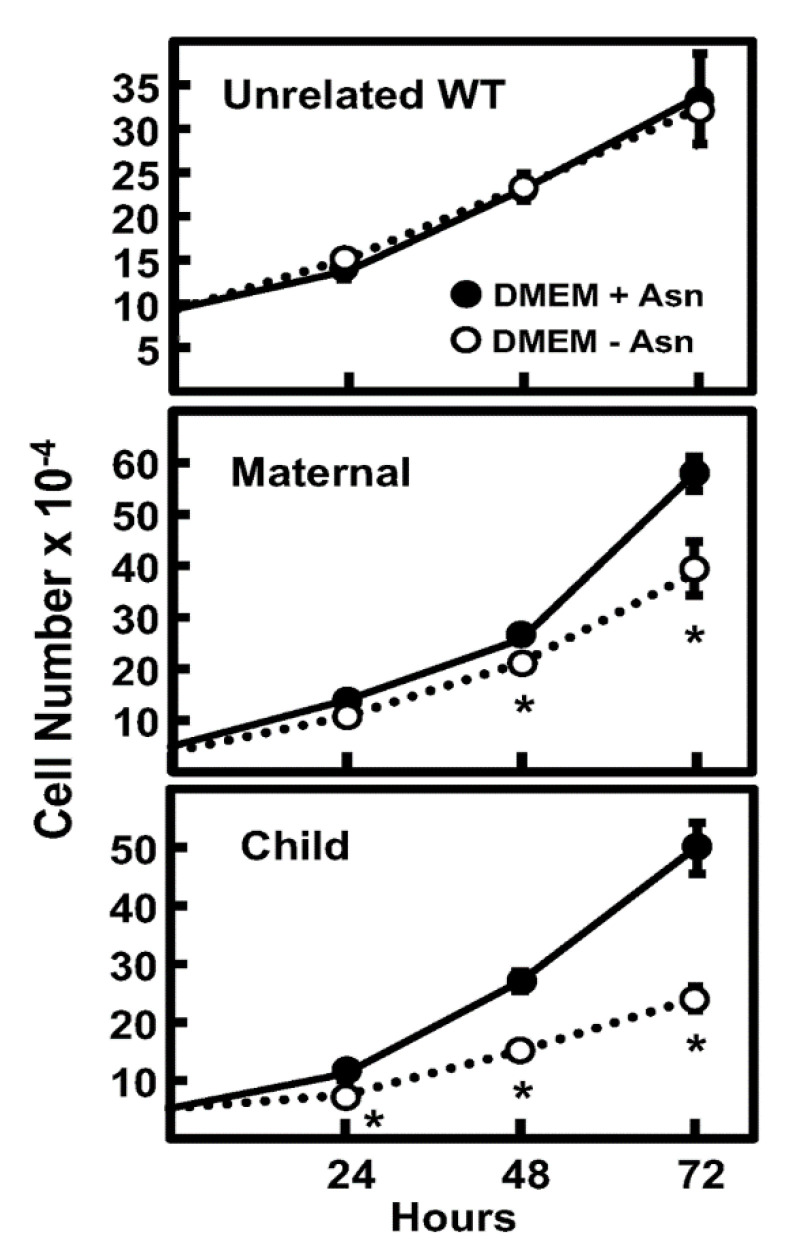
Cell proliferation of primary fibroblasts from mother and child with ASNS variants. The cell counts of WT, maternal, and child fibroblasts were measured from 0 to 72 h during incubation in DMEM ± Asn. The data are the averages ± standard deviations of three determinations, and an asterisk indicates that the DMEM - Asn value is statistically different from the DMEM + Asn condition with a *p*-value of ≤0.05.

The fibroblasts of the WT, mother, and child were cultured in DMEM medium with or without Asn for 72 h, and cell counts were determined every 24 h. The WT fibroblasts showed no significant difference in growth over the time measured, whereas both the maternal and child-derived cells showed a significant reduction in proliferation (Figure 5). At the 72 h timepoint, culture in the absence of medium-supplied Asn led to a 35% reduction in cell number for the mother and a 45% reduction for the child, relative to the condition with provided Asn (Figure 5). These data indicate that the N80S variant hinders cellular proliferation even in the presence of a WT allele, while the biallelic mutations that result in the expression of both N80S and S480F variants impede growth even further.

### 2.6. Analysis of Growth after Variant Expression in ASNS-Null JRS Cells

**Figure 6:**
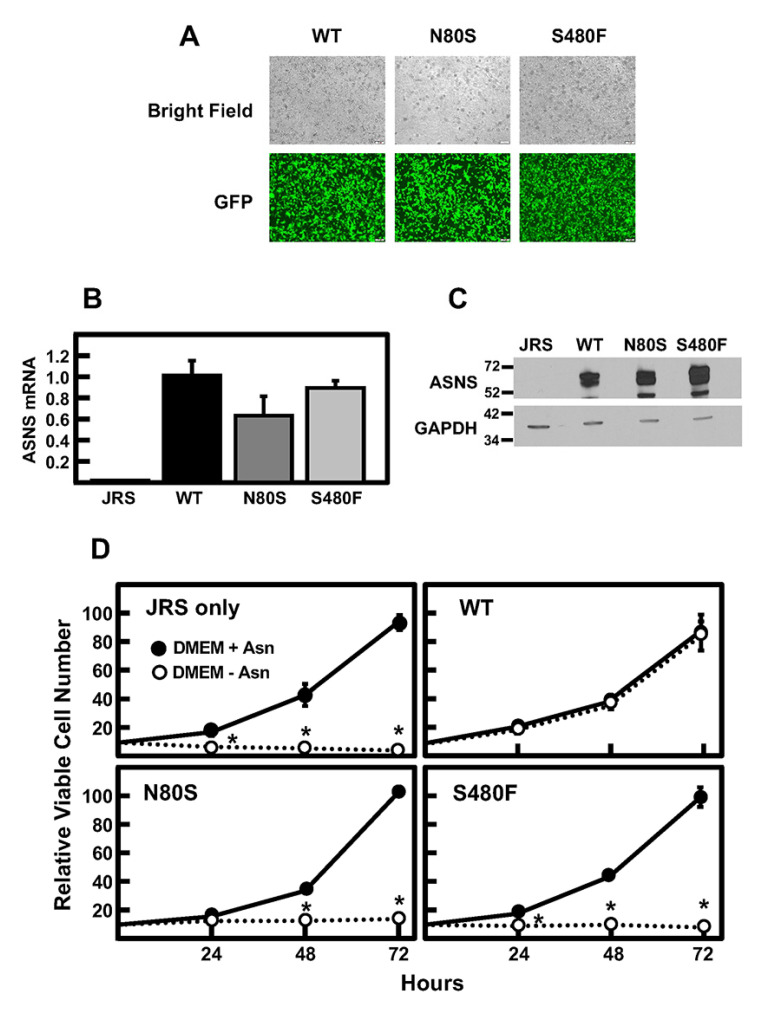
ASNS cDNA constructs encoding WT, N80S, and S480F were independently stably expressed in an ASNS-null background JRS cell line. (**A**) Co-expression of GFP with ASNS cDNA constructs permits evaluation of retroviral transduction efficiency in generation of the JRS model system. ASNS mRNA (**B**), as determined by qRT-PCR, and relative ASNS protein abundance (**C**) evaluated by immunoblotting for each of the generated JRS cell lines. (**D**) Cellular proliferation was determined by cell counting of the JRS cell lines in the presence or absence of Asn in the media for 0, 24, 48, and 72 h. The data are the averages ± standard deviations of three determinations, and an asterisk indicates that the DMEM—Asn value is statistically different from the Asn-containing condition with a *p*-value of ≤0.05.

To evaluate the impact on the growth of each variant, independent of the expression from another allele, a model system was developed using Jensen Rat Sarcoma (JRS) cells, which are ASNS-null due to hypermethylation of the *ASNS* gene [20]. Separate constructs for WT, N80S and S480F *ASNS* were stably expressed by retroviral transduction and selection of JRS cells, with a vector that co-expresses GFP as a reporter. Fluorescence imaging showed a high transduction efficiency for all three constructs (Figure 6A). To confirm ASNS expression, ASNS mRNA (Figure 6B) and protein (Figure 6C) were detected in each of the cell lines. Using these established cell lines, proliferation in the presence or absence of Asn was evaluated. As expected, JRS cells transduced with an empty vector failed to grow in the absence of medium-supplied Asn (Figure 6D). Conversely, ectopic expression of WT *ASNS* generated cells that showed no difference in proliferation between the +/− Asn conditions. In sharp contrast, however, neither cell line exclusively expressing the N80S or S480F ASNS variant was able to proliferate in the absence of Asn in the media. The JRS model system data provide functional evidence that neither of the ASNS variants expressed in the patient can contribute sufficient intracellular Asn production to maintain cell growth.

## 3. Discussion

ASNSD is a rare inborn error of metabolism first described in 2013 [10]. Some of the symptoms of this disease include the association of progressive epileptic encephalopathy, progressive microcephaly, and cortical blindness. The child presented here shows severe global developmental delay, progressive microcephaly, hyperreflexia, axial hypotonia followed by spastic quadriplegia, seizures, jitteriness, cortical blindness, thin corpus callosum, brain atrophy, and an unremarkable metabolic profile. This clinical presentation coincides with ASNSD cases previously reported in the literature [11,12,13].

Exome sequencing revealed that the child has biallelic mutations in the *ASNS* gene resulting in the expression of two protein variants, one containing an N80S substitution and another containing S480F. To our knowledge, the N80S variant is novel, whereas the S480F substitution has been reported previously [19]. The patient described by Gataullina et al. was also a compound heterozygote co-expressing a second variant containing an R550C substituted ASNS protein. Consequently, the authors were not able to evaluate the impact of the S480F variant independently. The present experiments measuring the enzymatic activity of ASNS protein with the S480F and ectopic expression of this variant in ASNS-null JRS cells serve to fill this gap in our knowledge of ASNSD-associated variants. Stable expression of S480F ASNS in HEK293T cells (for enzyme purification) and in JRS cells (for proliferation studies) revealed that the steady-state levels for mRNA and protein were similar to expressed WT protein. The immunoblot observations were particularly important for two reasons. First, this variant was inherited from the father, and we did not have the opportunity to collect his fibroblasts for protein analysis. Second, we had previously generated a protein model for ASNS containing the S480F substitution and suggested that the replacement of serine at position 480 with phenylalanine did not appear to cause major changes in the predicted protein structure, but the hydrophobic nature of phenylalanine relative to serine may lead to a change in solubility or stability [6]. Yet, the present results showing protein levels similar to WT protein after expression in both HEK293T and JRS cells suggest that the S480F variant is not highly unstable. The lack of a major change in the protein’s melting point relative to WT also supports this interpretation. However, the purification of the S480F variant from the established HEK293T cell line allowed assessment of enzymatic activity and revealed that the S480F variant has only 20% of the WT activity. Furthermore, ectopic expression of S480F in ASNS-null JRS cells allowed us to discover that when present alone, this variant cannot support cell proliferation in an Asn-free medium.

The evaluation of the novel N80S ASNS variant led to quite a different set of conclusions than those for S480F. The human crystal structure shows that residue N80 is located within the N-terminal half of the ASNS protein but is not directly within the glutaminase active site. N80 does appear to contribute to the N-terminal folding by hydrogen bonding to several nearby residues, and the substitution of serine at this position may result in the loss of these hydrogen bonds. We were able to obtain primary fibroblasts from the mother along with the child. Analysis of steady-state mRNA and protein in these cell lines revealed that whereas the child’s cells had ASNS expression similar to that in an unrelated WT fibroblast cell line, the cells from the mother exhibited a significant reduction in both mRNA and protein. The molecular basis for the reduction in ASNS mRNA and protein expression in the maternal fibroblasts will require further experimentation. If instability of the N80S variant contributes to the observed reduction in total ASNS protein, this effect appears to be cell context-specific in that N80S protein abundance was reduced relative to WT after expression in HEK293T cells but not after expression in JRS cells. Curiously, the abundance of N80S protein was clearly reduced in the parental fibroblasts and the HEK293T cells, both of which express some level of wild-type ASNS protein. In contrast, expression of the N80S variant in JRS cells, lacking wild-type ASNS, revealed no reduction in the steady-state level of the variant protein. This observation opens the possibility that the N80S variant becomes unstable in the presence of wild-type protein. Additional studies will be required to address the stability of the N80S variant. Despite the decreased cellular abundance after expression in HEK293T cells, purification of the N80S variant protein was readily achievable and revealed that the inherent enzymatic activity of this variant was reduced by about 50% relative to WT protein. Consistent with this decrease in enzymatic activity, when the N80S variant was expressed in ASNS-null JRS cells, there was little or no proliferation of the cells in the Asn-free medium.

Collectively, the cellular and biochemical analysis of the S480F (paternal) and N80S (maternal) ASNS variants document that both have significantly reduced enzymatic activity. For the heterozygotic parents, the existence of a WT allele appears to provide sufficient ASNS activity, along with dietary protein as a source of Asn, to permit growth and development. However, it should be noted that when challenged by culture in an Asn-free medium, growth of the maternal fibroblasts was partially suppressed. This cell-based observation is consistent with the 50% reduction of enzymatic function for the N80S variant expressed by the mother. Although fibroblasts were not available from the father, the 80% reduction of enzymatic activity of the purified S480F protein and its inability to support any cellular growth after expression in ASNS-null cells supports the conclusion that this amino acid substitution has considerable deleterious effects as well. Thus, the compound heterozygotic child appears to express two ASNS proteins, neither of which provides sufficient enzyme activity to support optimal cellular function. Consistent with this interpretation, the proliferation of the child’s fibroblasts maintained in an Asn-free medium was reduced by about 50% relative to growth in the presence of medium-supplied Asn.

Clearly, the common ASNSD features at birth, or shortly thereafter, of microcephaly, abnormal brain structures, and epileptic seizures indicate that Asn de novo synthesis within the brain is critical for normal development. A hypothesis has been put forth suggesting that the brain is particularly vulnerable in ASNSD children because of its reliance on de novo Asn synthesis rather than dietary Asn [6]. If correct, the neurological impairment of the child described in this report is consistent with the biallelic mutations that lead to ASNS variants with a 50% (N80S) and 80% (S480F) reduction in activity, respectively.

## 4. Materials and Methods

### 4.1. Exome Sequencing and Genetic Analysis

DNA was extracted from whole blood collected from the patient and her parents, and exome enrichment was performed by capture (SureSelect Human All Exon V6 + UTR kit, Agilent, Santa Clara, CA, USA). Exome sequencing was performed for the proband on the CNRGH platform in Evry, France using a HiSeq 4000 system (Illumina, San Diego, CA, USA). Alignment to the reference genome (build GRCh37/hg19) was conducted using the BWA tool [21]. Detection of sequence variations and realignment around indels was performed with GATK (v.0.7.6a-3.6.0) [22] and Picard (http://broadinstitute.github.io/picard, v1.141) tools and annotation by SnpEff [23]. Copy number variations were established by the CANOES tool based on the sequencing depth profile [24]. Variations were filtered according to the modes of transmission: de novo autosomal dominant, autosomal dominant, autosomal recessive, and X-linked; and interpretation of variations was conducted according to the recommendations of the American College of Medical Genetics and Genomics and the Association for Medical Pathology [25]. Variations of diagnostic interest were independently confirmed by Sanger sequencing. The primers used for amplification and Sanger sequencing for exon 3 of *ASNS* were 5′-**TCGGATAGTCAGTCGTTT**TGACCAAGTCATTTCACTCACCT-3′ (forward) and 5′ **ACCGTTAGTATGCGAGTT**CCATGTGTGGCATTTGGGC-3′ (reverse), and 5′**TCGGATAGTCAGTCGTTT**ACAGCAATGACAGCTCTGCA-3′ (forward) and 5′-**ACCGTTAGTATGCGAGTT**ATCAACTCAAGGTAAAACTGTCCA-3′ (reverse) for exon 12. Universal primers were used (in bold).

### 4.2. Protein Modeling

Using the UCSF Chimera software (version 1.15), the human ASNS structure (PDB: 6GQ3) [7] was overlayed with the *E. Coli* AS-B structure (PDB: 1CT9) [8], allowing for human ASNS modeling with glutamine bound in the N-terminal human ASNS structure and AMP bound in the C-terminal *E. Coli* AS-B structure. N80S and S480F variants were introduced with the rotamers tool, and the most probable rotamer was selected based on backbone-dependent rotamer library predictions [26]. Predictions of hydrogen bonding were performed with the FindHBond tool, and predictions of steric interference were obtained through the Find Clashes tool in the Chimera software.

### 4.3. Cell Culture

Primary fibroblast cell lines were generated from skin biopsies taken from the affected child and the mother. As a wild-type (WT) control, an unrelated human dermal fibroblast cell line was obtained from ATCC (#PCS-201-012), as were HEK293T cells (#CRL-3216) and Jensen Rat Sarcoma (JRS) cells (#CCL-45). All cell lines were cultured in high glucose Dulbecco’s Modified Eagle’s Medium (DMEM) (Corning, Tewskbury, MA, USA, #10-013-CV) supplemented with 10% fetal bovine serum (FBS, Bio-Techne, Minneapolis, MN, USA, #S11550), 1X ABAM (streptomycin, penicillin G, and amphotericin B), 1X non-essential amino acids, and 2 mM glutamine. DMEM without Asn was prepared by using 10% dialyzed FBS (Bio-Techne #S12850) and replacing the 1X non-essential amino acids supplement with 0.1 mM glycine, L-alanine, L-aspartate, L-glutamate, L-proline, and L-serine. For experiments lasting longer than 24 h, fresh medium was supplied every 24 h.

### 4.4. RNA Purification and Quantitative RT-PCR

Total RNA was isolated using TRIzol Reagent (Invitrogen, Waltham, MA, USA, #15596026) and converted to cDNA with the qScript cDNA synthesis kit (Quantabio, Beverly, MA, USA, #95047). The qRT-PCR reaction mixture contained cDNA, 300 nM forward and reverse primers, and SYBR Green PCR Master Mix (Applied Biosystems, Waltham, MA, USA, #4309155), and the reaction was performed in a Bio-Rad CFX-Connect Real-Time System. The cycling parameters were: 1 × (95 °C for 10 min), 40 × (95 °C for 15 s and 60 °C for 1 min with an SYBR measurement), 1 × 95 °C for 15 s, and a melting curve from 55 °C to 98 °C increasing 0.5 °C every 5 s. Primers used for ASNS mRNA were forward 5′-GCAGCTGAAAGAAGCCCAAGT-3′ and reverse 5′-TGTCTTCCATGCCAATTGCA-3′ and those used for GAPDH mRNA were forward 5′-TTGGTATCGTGGAAGGACTC-3′ and reverse 5′-ACAGTCTTCTGGGTGGCAGT-3′. Analysis of the data was performed by the ∆∆Ct method [27].

### 4.5. Immunoblotting

Cells were resuspended in RIPA buffer (Boston BioProducts, Milford, MA, USA, #BP-115D) with 1× protease inhibitors (Roche, Basel, Switzerland, #04693159001). Resuspended pellets were incubated for 15 min at 4 °C, and then cell debris was removed by centrifugation at 13,000× *g* for 15 min. A Bradford assay (Bio-Rad, Hercules, CA, USA, #5000006) was used to measure protein content, and 30 μg of protein was loaded per lane. Electrophoresis and immunoblotting were performed as described previously [17]. Antibody dilutions were: 1:200 ASNS monoclonal antibody [28], 1:1000 FLAG primary antibody (Cell Signaling, Danvers, MA, USA, #147935), 1:5000 anti-mouse HRP antibody (Bio-Rad #170-6516), and 1:10,000 anti-rabbit HRP-conjugated antibody (Bio-Rad, Hercules, CA, USA, #170-6515).

### 4.6. Cell Growth Analysis

Primary fibroblasts were plated at 5 × 10^4^ cells per well in 6-well plates and then incubated in DMEM ± Asn for 72 h, with fresh media supplied every 24 h. To analyze cell numbers, the wells were rinsed with ice-cold PBS to remove dead cells and debris; the remaining cells were detached with 0.25% trypsin and collected by centrifugation at 300× *g* for 5 min. Viable cell counts were determined using the Applied Biosystems Countess II FL automated cell counter per the manufacturer’s trypan blue staining protocol. The percentage of viable cells was routinely greater than 95%.

### 4.7. Purification of ASNS Protein by FLAG Immunoprecipitation

A commercially available C-terminal FLAG-tagged WT ASNS expression construct (Sino Biological (#HG16454-CF) was used to generate mutated constructs encoding 239A > G (N80S) and 1439C > T (S480F) by site-directed mutagenesis from Genscript. Using X-tremeGENE 9 transfection reagent (Roche, Basel, Switzerland, #06 365 787 001), each of these constructs was used to transfect HEK293T cells. Stable lines were selected over 14 d in DMEM containing 100 μg/mL Hygromycin B (EMD Millipore, Burlington, MA, USA, #400052-5ML) and then maintained in the presence of 50 μg/mL of hygromycin B. ASNS-FLAG expression was confirmed by immunoblotting with anti-FLAG antibody (Cell Signaling #147935). FLAG-tagged ASNS proteins were purified as described [17]. Protein purity was assessed with SDS-PAGE and Coomassie Brilliant Blue R-250 staining (Bio-Rad 1#61-0400). Single-use aliquots were stored at −80 °C and thawed only once.

### 4.8. AMP-Based ASNS Enzyme Assay

The Promega AMP-Glo Assay (#V5011) kit was used to measure AMP production by purified ASNS protein. In brief, a 100 ng aliquot of ASNS protein was incubated with 50 mM Tris, 10 mM L-glutamine, 10 mM L-aspartate, 1 mM ATP, 0.75 mM DTT, 0.05 mg/mL BSA, and 10 mM MgCl_2_ for 30 min at room temperature in a 25 μL final volume. The final enzyme concentration was 62.5 nM, and the BSA was included to prevent adherence of the ASNS protein to the reaction vessel. To prevent excess ATP interference with AMP determination, the reaction was then diluted 1:10 with 50 mM Tris to lower the residual ATP concentration, and 25 μL of this dilution was used to measure AMP per the manufacturer’s protocol. Assays were performed in triplicate to test variability, and experiments were repeated at least once with independent protein preparations to establish reproducibility. Significant differences between WT and variant ASNS proteins were determined with the Student’s *t*-test.

### 4.9. Differential Scanning Fluorimetry (DSF) for Thermal Stability Assay

The melting point of either WT, N80S or S480F ASNS was determined with 2.5 μg of purified protein, 2.5 μL of 50× SYPRO Orange (Invitrogen, Waltham, MA, USA, #S6651 diluted to 50×), and up to a final volume of 25 μL with Tris Buffered Saline (TBS, 30 mM Tris pH 7.6, 200 mM NaCl). These samples were analyzed in Bio-Rad CFX-Connect Real-Time System with cycling parameters set to increment from 10 °C to 95 °C with increments of 0.5 °C per 10 s and fluorescence detection after each increment.

### 4.10. Retroviral Transduction of Jensen Rat Sarcoma Cells

Genscript (Piscataway, NJ, USA, was used to clone the ASNS-FLAG open reading frame from the C-terminal FLAG-tagged ASNS expression construct (Sino Biological, Bejing, China, #HG16454-CF) between the BglII and XhoI restriction sites of the MSCV-PIG (Puro IRS GFP) expression plasmid (Addgene, Watertown, MA, USA, #18751). The resulting ASNS-FLAG MSCV-PIG (Puro IRES GFP) sequence was then mutated by Genscript to generate independent 239A > G and 1439C > T mutant ASNS sequences. The MSCV-PIG plasmid codes for puromycin selection and an IRES-driven green fluorescence protein (GFP). Retrovirus packaging in HEK293T cells and viral infection of Jensen rat sarcoma cells (JRS) have been described previously [17]. The efficiency of transduction was confirmed by GFP fluorescence and ASNS expression by immunoblotting.

### 4.11. Statistical Analysis

The analytical assays were in triplicate for a given experiment to determine the variability, and all experiments were repeated at least once with an independent batch of cells to assess reproducibility. The data reported are the averages ± standard deviations and were analyzed by Student’s two-tailed *t*-test. Where indicated with an asterisk, the differences have a *p*-value of ≤ 0.05. For immunoblots and GFP staining, the data represent individual experiments, which were repeated at least once to ensure reproducibility.

## Data Availability

All original data will be made available upon request.

## Abbreviations

Asn	Asparagine
ASNSD	Asparagine synthetase deficiency
ASNS	Asparagine synthetase
GAPDH	Glyceraldehyde-3-phosphate dehydrogenase
PDB	Protein database
SD	Standard deviation
